# The bigger, the better? Volume measurements of parasites and hosts: Parasitic barnacles (Cirripedia, Rhizocephala) and their decapod hosts

**DOI:** 10.1371/journal.pone.0179958

**Published:** 2017-07-05

**Authors:** Christina Nagler, Marie K. Hörnig, Joachim T. Haug, Christoph Noever, Jens T. Høeg, Henrik Glenner

**Affiliations:** 1 Department of Biology II, Ludwig-Maximilians University of Munich, Planegg-Martinsried, Germany; 2 Cytology and Evolutionary Biology, Ernst-Moritz-Arndt University of Greifswald, Greifswald, Germany; 3 GeoBio-Center, Ludwig-Maximilians University of Munich, Munich, Germany; 4 Department of Marine Biology, University of Bergen, Bergen, Norway; 5 Marine Biology Section, Department of Biology, University of Copenhagen, Copenhagen, Denmark; CNRS, FRANCE

## Abstract

Rhizocephala, a group of parasitic castrators of other crustaceans, shows remarkable morphological adaptations to their lifestyle. The adult female parasite consists of a body that can be differentiated into two distinct regions: a sac-like structure containing the reproductive organs (the externa), and a trophic, root like system situated inside the hosts body (the interna). Parasitism results in the castration of their hosts, achieved by absorbing the entire reproductive energy of the host. Thus, the ratio of the host and parasite sizes is crucial for the understanding of the parasite’s energetic cost. Using advanced imaging methods (micro-CT in conjunction with 3D modeling), we measured the volume of parasitic structures (externa, interna, egg mass, egg number, visceral mass) and the volume of the entire host. Our results show positive correlations between the volume of (1) entire rhizocephalan (externa + interna) and host body, (2) rhizocephalan externa and host body, (3) rhizocephalan visceral mass and rhizocephalan body, (4) egg mass and rhizocephalan externa, (5) rhizocephalan egg mass and their egg number. Comparing the rhizocephalan *Sylon hippolytes*, a parasite of caridean shrimps, and representatives of *Peltogaster*, parasites of hermit crabs, we could match their different traits on a reconstructed relationship. With this study we add new and significant information to our global understanding of the evolution of parasitic castrators, of interactions between a parasitic castrator and its host and of different parasitic strategies within parasitic castrators exemplified by rhizocephalans.

## Introduction

### Parasitism in crustaceans

Rhizocephalan parasites (Crustacea, Cirripedia) exhibit one of the most extremely divergent forms of parasites in animals [1]. Although, they are crustaceans, the adults have lost virtually all vestiges of their crustacean ancestry [2, 3]. The body has two parts [4–7]: a) a sac-like, reproductive structure (externa) protruding from the cuticle of their crustacean host, and b) a nutrient-absorbing rootlet system (interna) that spreads through the host’s body, similar to a fungal rhizome. The ramifying complexity of the interna has made it difficult to quantify the size (mass) of the parasite relative to its host. We overcome this challenge by using advanced, non-invasive micro-CT imaging and 3D reconstruction to estimate, for the first time, body sizes of parasites and their hosts, and potential reproductive output of these parasites.

### Akentrogonid and Kentrogonid lifestyle

**Fig 1:**
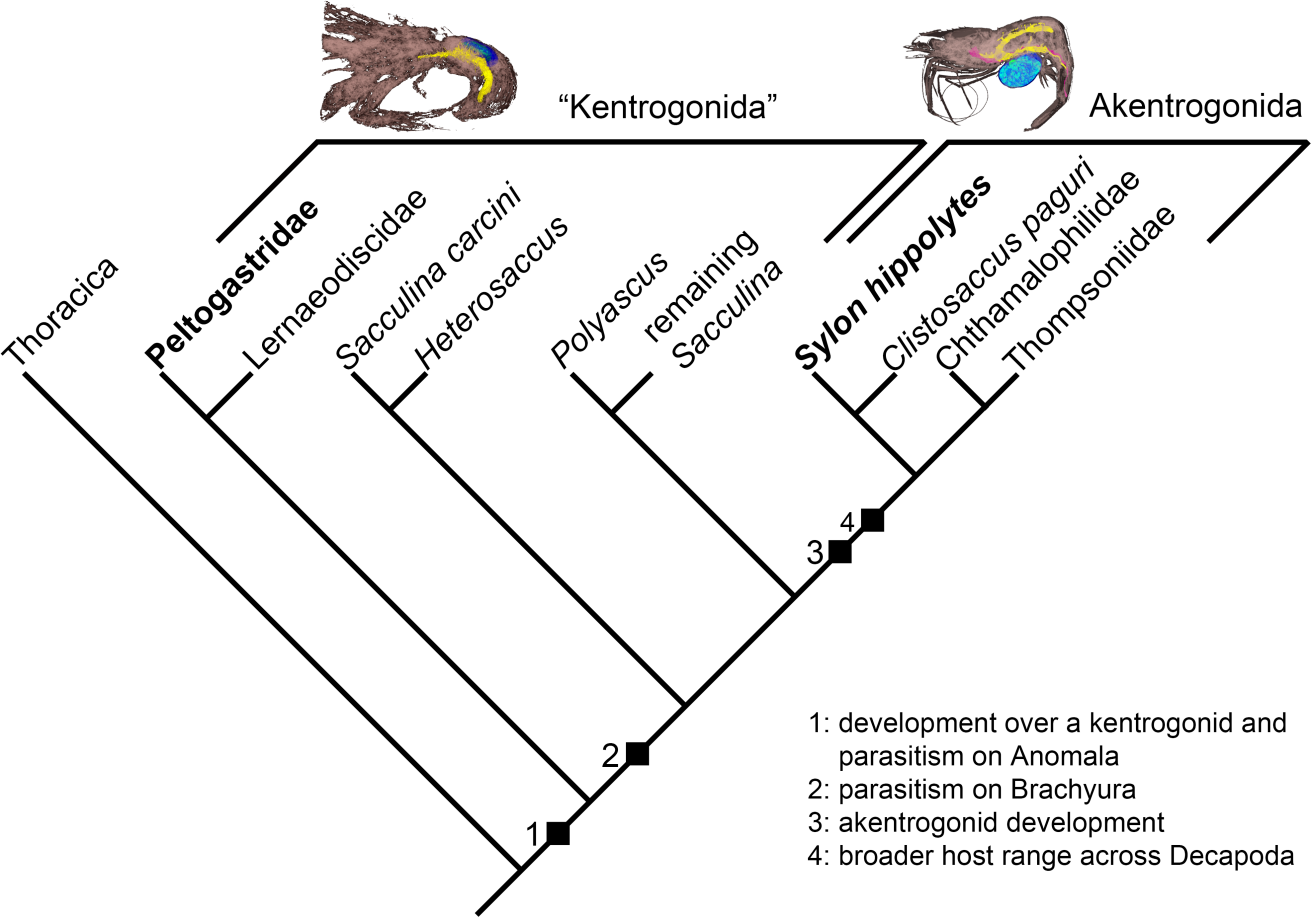
Reconstructed relationship of Rhizocephala with outgroup Thoracica after [1, 5]. Note the paraphyly of “Kentrogonida” with a monophyletic Akentrogonida. For further information, please follow the discussion in [1, 5].

Due to their different life cycles, Rhizocephala can be divided into forms that are developing with a kentrogon stage (“Kentrogonida”) and forms without a kentrogon in their larval development (Akentrogonida) (Fig 1; [5, 8]). In kentrogonid rhizocephalans the nauplius larvae develop through several stages and eventually reach the cypris stage. Females form a so-called kentrogon that inject a tiny cuticle-clad structure of itself (called vermigon) into the hemolymph of their host [9]. The vermigon grows into an extensive internal root system and when it has reached a certain size, the virgin externa develops, which contains the reproductive apparatus of the parasite. Male cypris larvae settle on the virgin externa and implant the trichogon stage into one of the receptacles provided by the virgin externa [8, 10].

In contrast, akentrogonids have lost the naupliar phase and the kentrogon or trichogon stage. The female cypris injects the internal parasite directly. After development of a root system and the externa, a male cypris larva penetrates the integument of the externa and injects spermatogonia cells into the receptacles of the externa [8, 11].

Based on molecular data [1, 5] monophyletic Akentrogonida and paraphyletic “Kentrogonida” form together Rhizocephala (Fig 1). The earliest branch, within kentrogonid rhizocephalans, contains representatives of Peltogastridae, while the widely-known Sacculinidae, common parasites of brachyuran crabs, have evolved more recently [5]. Based on molecular and morphological analyses, *S*. *hippolytes* has been suggested to form a derived group together with *Clistosaccus paguri* within Akentrogonida [1].

### Rhizocephala as parasitic castrators

Rhizocephalans are among the three percent of crustaceans that are obligatory parasitic castrators of other crustaceans [12]. The castrating or sterilizing interaction between consumer (parasite) and resource (host) is unique among parasitic strategies [13]. Parasitic castrators suppress or prevent host reproduction, but, in contrast to parasitoids, do not kill their hosts [14]. Often parasitic castrators change the behavior and metabolism of their hosts, e.g. some rhizocephalans suppress molting in crustaceans [13]. Apparently, animals with high reproductive effort and a relatively long adult life–particularly decapods–seem to be the preferred host of parasitic castrators, because the parasitic castrator absorbs the entire reproductive energy and occupies the space of reproductive organs of the host. Thus, the combination of high reproductive effort and long life span makes castration profitable in comparison to other consuming strategies [13, 15]. The compromise between feeding and longevity of the parasite and the reproductive death of the host results in this incomparable relation between parasite and host in parasitic castrators [13]. Thus, the size of the host and parasite and their ratio is crucial to the nature of this relationship [16].

Using modern imaging methods such as micro-CT, a three-dimensional, non-invasive view of a rhizocephalan parasite and its crustacean host is possible [7]. Due to the difficulties quantifying the size of rhizocephalans in relation to their hosts, this study aims at quantifying the volume and size of rhizocephalans and their hosts by such a non-invasive approach using micro-CT. Based on reconstructed models of parasites and hosts using different grey values for reconstruction, measurement of the volumes is feasible. In this study we present for the first time volume measurements of a rhizocephalan exemplified by four species of *Peltogaster*, parasitic on hermit crabs and another five specimens of *S*. *hippolytes* parasitic on shrimps. Furthermore, this study aims at evaluating different life history traits linked to the two groups among rhizocephalans, Kentrogonida and Akentrogonida. The presented results show differences in the reproduction and life span of Akentrogonida and Kentrogonida. These differences could be mapped on their phylogenetic tree.

## Material and methods

### Material of *Sylon hippolytes*

We collected 22 specimens of the shrimp *Pandalina brevirostris* (Rathke, 1843) infested with the rhizocephalan *S*. *hippolytes* Sars, 1870 during a sampling cruise in October 2015 with the research vessel Hans Brattström in the Hjeltefjord, near Bergen, Norway.

**Fig 2:**
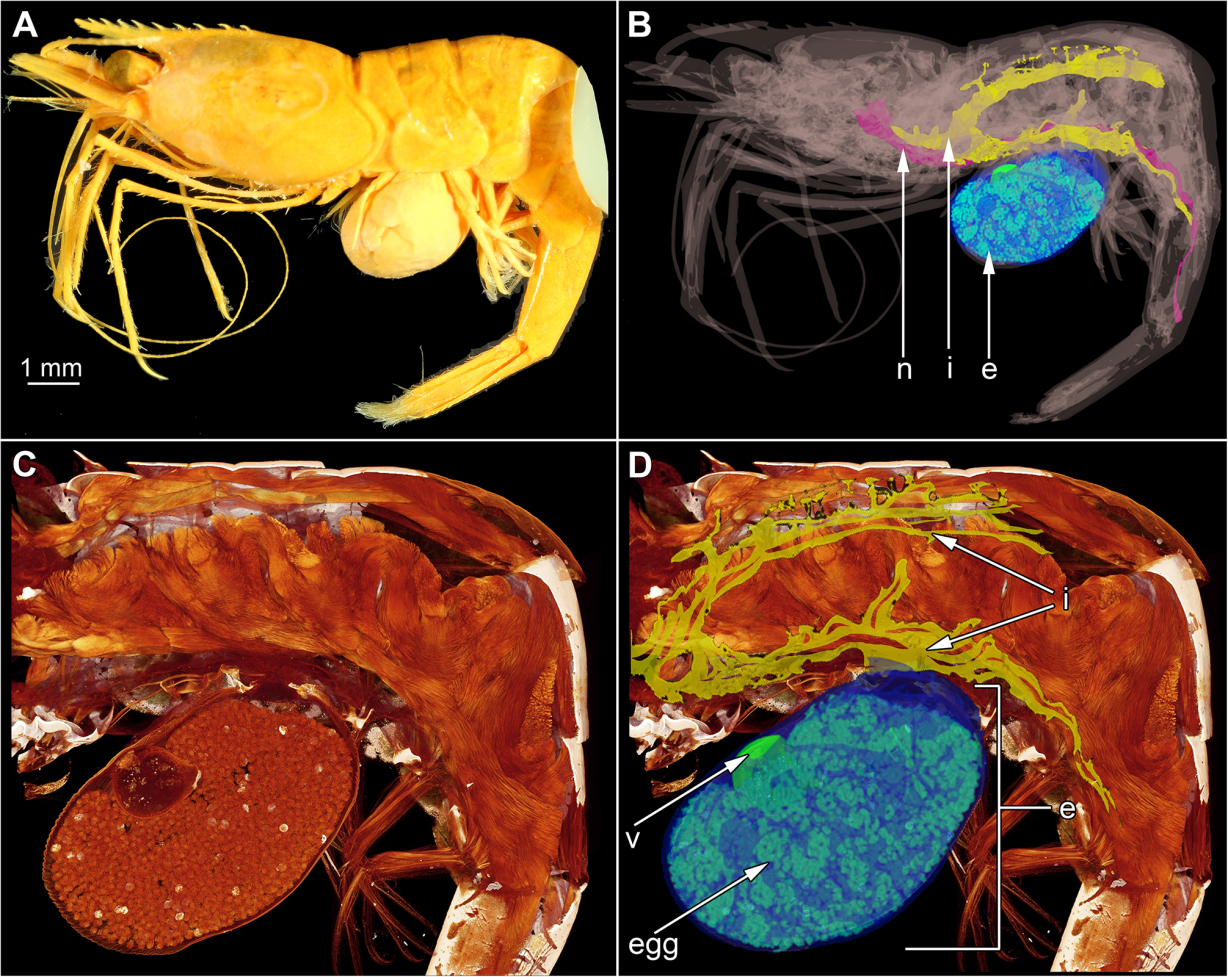
Macro-photograph, surface model and volume rendering of *Sylon hippolytes* infesting *Pandalina brevirostris* (specimen 1b). Color-markings: *P*. *brevirostris* = grey, nervous system (n) of *P*. *brevirostris* = pink, interna (i) of *S*. *hippolytes* = yellow, externa (e) of *S*. *hippolytes* = blue, eggs of *S*. *hippolytes* = turquoise, visceral mass of *S*. *hippolytes* = green. (A) Macro-photograph. Scale = 1 mm. (B) Surface model. (C) Detail of volume rendering. (D) Same as C with corresponding surface model.

**Fig 3:**
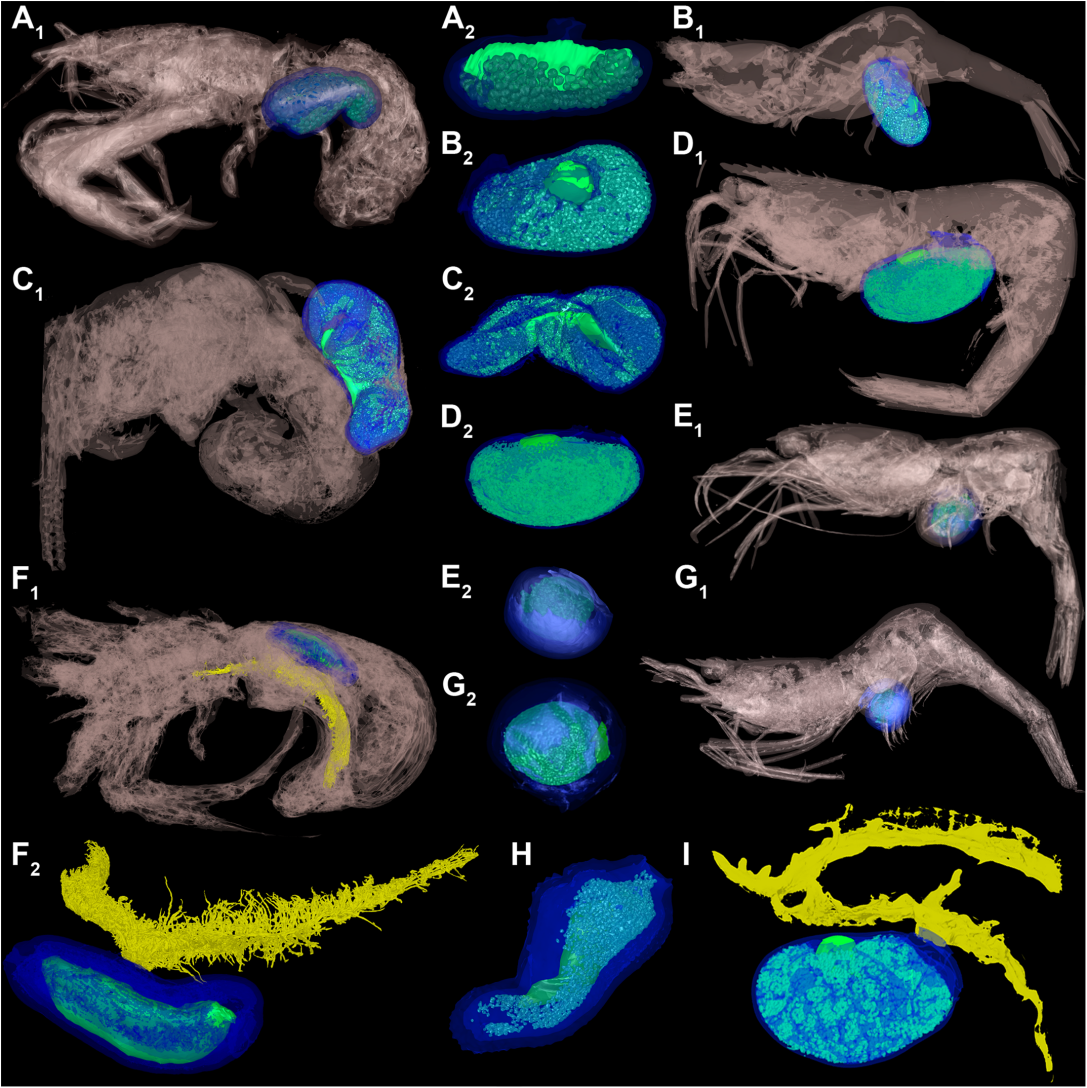
Surface models of all specimens investigated herein. *Peltogaster* spp. infesting different hermit crabs (A, C, F, H), *Sylon* spp. infesting *Pandalina brevirostris* (B, D, E, G, I). Color-markings: host = grey, rhizocephalan externa = blue, rhizocephalan interna = yellow, rhizocephalan eggs = turquoise, rhizocephalan visceral mass = green. Not to scale. (A) *P*. *boschmai* with detail of respective externa. (B) *S*. *hippolytes* specimen 2b with detail of respective externa. (C) *Peltogaster* sp. 1 with detail of respective externa. (D) *S*. *hippolytes* specimen 2a with detail of respective externa. (E) *S*. *hippolytes* specimen 1c with detail of respective externa. (F) *Peltogaster* sp. 2 with detail of respective externa. (G) *S*. *hippolytes* specimen 1a with detail of respective externa. (H) Externa of *P*. *curvata*. (I) *S*. *hippolytes* specimen 1b.

We sampled two times with a benthic sledge after Rothlisberg and Pearcy [17] between N 60°37.567, E 004°52.479 (209 m depth) and N 60° 37.056, E 004°53.031 (224 m depth). The sledge was pulled for 15 minutes over the ground on a 1070 m long steel rope. The first tow showed an infestation rate of 24% (eight of 33 *P*. *brevirostris* were infested with *S*. *hippolytes*) (Specimen 1a-c, Figs 2 and 3E, 3G and 3I). The second tow showed an infestation rate of 27% (14 of 52 *P*. *brevirostris* were infested with *S*. *hippolytes*) (Specimen 2a-b, Fig 3B and 3D). Samples were fixed during the campaign in 4% Para-formaldehyde in phosphate buffered saline.

### Material of *Peltogaster* spp. for comparison

Four specimen of the hermit crab-infesting *Peltogaster* were included in the analyses. The preparation of the *Peltogaster* material is described in Noever et al. [7]. The following species were studied: *Peltogaster curvata* Kossmann, 1874 infesting the hermit crab *Pagurus prideaux* Leach, 1815 from Western Norway (Fig 3H), *Peltogaster boschmai* Reinhard, 1944 infesting the hermit crab *Discorsopagurus schmitti* (Stevens, 1925) from Washington State, USA (Fig 3A), *Peltogaster* sp. 1 infesting the hermit crab *Pagurus hirsutiusculus* (Dana, 1851) from Southeastern Alaska, USA (Fig 3C) and *Peltogaster* sp. 2 infesting the hermit crab *Pagurus pubescens* Krøyer, 1838 from the Svalbard Archipelago, Norway (Fig 3F).

### Preparation of *S*. *hippolytes* for micro-CT

#### Staining with iodide

After transferring two specimens from each tow (*S*. *hippolytes* specimen 1a & 2a) via a gradual ethanol sequence (10% EtOH, 30% EtOH, 40% EtOH, 50% EtOH, 60% EtOH, 70% EtOH, 80% EtOH, 90% EtOH, 96% EtOH, each for 1 day) in absolute ethanol, they were stained with 1% iodine over night.

In the externa of one specimen (*S*. *hippolytes* specimen 1b & 2b) of each tow, we injected 0.5 ml 2% iodine in absolute ethanol for two hours. After washing the specimens in absolute ethanol (2x20 min), they were critical point dried with a Polaron E3100 (Quorum Technologies, Lewes, England) in the Laboratory for Electron microscopy of the University of Bergen (Norway).

#### Staining with phosphotungstid acid

After transferring one specimen from each tow (*S*. *hippolytes* specimen 1c & 2c) through a gradual ethanol sequence (10% EtOH, 30% EtOH, 40% EtOH, 50% EtOH, 60% EtOH each for 1 day) in 70% ethanol, they were stained with 1% iodine over night. The specimens were washed in 70% ethanol (2x 20 min), transferred to 70% ethanol. We injected 0.5 ml 1% phosphotungstid acid (PTA) in the externa of the specimen after Metscher [18].

### Documentation

Five specimen of *S*. *hippolytes* were documented with macro photography and x-ray micro-CT scanning (Specimen 1a: Fig 3G, specimen 1b: Figs 2A–2E and 3I, specimen 1c: Fig 3E, specimen 2a: Fig 3D, specimen 2b: Fig 3B).

Macro-photography (combined with composite imaging) was performed following [19–21] under cross-polarized light. We used a Canon EOS Rebel T3i camera, either with a Canon EFS (18–55 mm) lens (for overview images) or a Canon MP-E (65 mm) macro lens (for detail images). Illumination was provided by a Canon Macro Twin Lite MT-24EX flash from the two opposing sites.

Stacks of images were processed with the freeware packages CombineZP (Alan Hadley), ImageAnalyzer (Meesoft) and ImageJ (Wayne Rasband). Assembling of stereo images and final processing (levels, sharpness, and saturation) was performed in Adobe Photoshop CS4.

Micro-CT of every specimen of *S*. *hippolytes* was performed with XRadia XCT-200 (Carl Zeiss Microscopy GmbH, Jena, Germany) equipped with switchable scintillator objective lens units (details see [22, 23]). *S*. *hippolytes* specimen 1a-b and specimen 2a-b were scanned in air, *S*. *hippolytes* 1c was scanned in 70% EtOH. Tomography was performed using magnifications of 0.39x and 4x objectives. X-ray source setting was: (1) overview scans: 30 kV and 6 W for 2 s (*S*. *hippolytes* specimen 1b), 3 s (*S*. *hippolytes* specimen 2a-b), and respectively 4 s (*S*. *hippolytes* specimen 1a, c) acquisition time; (2) detail scan: 40 kV and 8 W for 5 s (*S*. *hippolytes* specimen 1b). Image stack properties were: (1) overview scans: calculated pixel size = 16.64 μm (*S*. *hippolytes* specimen 1a), 12.62 μm (*S*. *hippolytes* specimen 1b), 19.65 μm (*S*. *hippolytes* specimen 1c), 15.39 μm; 1024 x 1024 px; (*S*. *hippolytes* specimen 2a-b) detail scan: calculated pixel size = 5.54 μm, 1015 x 1015 px. Tomography projections were reconstructed using the XMReconstructor software (Carl Zeiss Microscopy GmbH, Jena, Germany), resulting in image stacks (TIFF format). All scans were performed using Binning 2 and subsequently reconstructed using Binning 1 (full resolution) to avoid information loss. The resulting image stacks were processed with ImageJ and volume renderings were generated using Amira 5.6 (FEI, Hillsboro, OR, USA).

### Measurements

Tiff stacks were further processed with ImageJ (Wayne Rasband) and Osirix 5.8.2 (Antoine Rosset). Surface models created (‘segmented’ or by thresholds over the grey values) in Osirix were further modified with Blender 2.49 (Blender Foundation). Due to the contrast given by the CT in the specimens in which iodine was injected, it was possible to reconstruct the interna of *S*. *hippolytes* specimen 1b (Figs 1B, 1D and 2I) via a threshold of the grey value. Volume measurements were calculated with the ‘3D-printing toolbox’ in Blender 2.67 (Blender Foundation). We calculated:

1. Volume of the surface model of the host (Vol_H_) for *P*. *pubescens*, *P*. *hirsutiusculus*, *D*. *schmitti*, *P*. *prideaux* and *P*. *brevirostris* specimen 1a-c and specimen 2a-b.
2. Volume of the surface models of the ‘interna’ (parasite tissue inside the host tissue) (Vol_I_) of *Pagurus* sp. 2 and *S*. *hippolytes* specimen 1b.
3. Volume of the surface models of the ‘externa’ (parasite tissue outside the host tissue) (Vol_E_) of *P*. *curvata*, *P*. *boschmai*, *Peltogaster* sp. 1, *Peltogaster* sp. 2 and *S*. *hippolytes* specimen 1a-c and specimen 2a-b.
4. Volume of the surface models of the ‘visceral mass’ (ovaries and receptacles) (Vol_V_) of *P*. *curvata*, *P*. *boschmai*, *Peltogaster* sp. 1, *Peltogaster* sp. 2 and *S*. *hippolytes* specimen 1a-b and specimen 2a-b.
5. Volume of the surface models of the ‘egg mass’ (Vol_Egg_) of *P*. *curvata*, *P*. *boschmai*, *Peltogaster* sp. 1 and *S*. *hippolytes* specimen 1a-c and specimen 2a-b.
6. Volume of an average egg (by measuring the volume of ten individual eggs and calculating the mean value) (Vol_AE_) of *P*. *curvata*, *P*. *boschmai*, *Peltogaster* sp. 1 and *S*. *hippolytes* specimen 1a-c and specimen 2a-b.
7. The number of eggs were either estimated by dividing the volume of the egg mass by the volume of an average egg (N_EE_) or counted with the 3D-object-counter plug-in (N_CE_) in ImageJ according to Bolte & Cordeliéres [24].

The measured volumes are relative values with artificial units, because the focus of this study lies on the relation between the parasite`s and host`s volume and not on the absolute value of them. Furthermore, the program Blender measures the volume in cm^3^, we cannot offer these values, because they are calculated with a default voxel size. Due to missing voxel size for different scans, we introduce ‘artificial units’. Artificial units of Vol_H_, Vol_E_, Vol_Egg_, Vol_V_ were calculated by the measured volume divided by 10^6^. Thus, we present the ratios between (1) the parasite (externa + interna) and the host, (2) the externa and the host, (3) the externa and the interna, (4) the visceral mass and the externa, (5) a single egg and the host and (6) a single egg and the entire egg mass.

We calculated the mean values and standard deviation of the ratios Vol_V_/Vol_E_, Vol_E_/Vol_H_, Vol_AE_/Vol_H_ and of N_EE_ and N_CE_. Shown is the mean value ± standard deviation. Statistical significances are indicated as asterisks determined by Student’s t-test: *, **, *** for p<0.05, p<0.01 and p<0.001, respectively, for the number of estimated and counted eggs and for the ratio between the volume of the externa and the volume of the host.

## Results

There was no visceral mass visible in *S*. *hippolytes* 1c and *Peltogaster* sp. 2 carried no eggs.

### Volume ratio between the parasite and the host

Vol_P_/Vol_H_ and Vol_I_/Vol_H_ are similar for *Peltogaster* sp. 2 and *S*. *hippolytes* specimen 1b in their respective hosts (Table 1). The average ratio Vol_E_/Vol_H_ does not differ significantly between *Peltogaster* and *S*. *hippolytes* (Table 2, p = 0.48).

**Table 1:** Ratio of the volumes of the surface models of the rhizocephalan parasite, externa, interna and the host. The following parameters are results for one individual of each species, therefore, no error estimate is possible.

Parasite	Host	Vol_P_/Vol_H_	Vol_I_/Vol_H_	Vol_I_/Vol_E_
*Peltogaster* sp. 2	*P*. *pubescencs*	17.78%	4.54%	34.24%
*S*. *hippolytes* 1b	*P*. *brevirostris*	18.07%	2.97%	18.82%

**Table 2:** Ratio of the volumes of the surface models of the rhizocephalans externa, visceral mass, and the host. The following parameters are results for four individual specimen of *Peltogaster* and five individual specimen of *Sylon*, therefore, no error estimate is possible for each individual.

Specimen	Host	Vol_E_/Vol_H_	Vol_E_/Vol_H_ (mean ± standard devation)		Vol_V_/Vol_E_	Vol_V_/Vol_E_ (mean ± standard deviation)	
*P*. *boschmai*	*D*. *schmitti*	12.38%	12.08 ± 2.44%	12.78 ± 2.11%	17.70%	19.08 ± 13.67%	12.50 ± 4.56%
*P*. *curvata*	*P*. *prideaux*	10.20%			10.45%		
*Peltogaster sp*. *1*	*P*. *hirsutiusculus*	15.29%			9.33%		
*Peltogaster sp*. *2*	*P*. *pubescens*	13.25%			38.82%		-
*S*. *hippolytes* 1a	*P*. *brevirostris*	10.95%		11.52 ± 2.78%	2.02%	2.48 ± 0.42%	-
*S*. *hippolytes* 2a		11.76%			2.84%		
*S*. *hippolytes* 1b		15.61%			2.83%		
*S*. *hippolytes* 2b		11.52			2.21%		
*S*. *hippolytes* 1c		7.82%			-	-	

### Volume ratio within the parasite between externa, interna, egg mass and visceral mass

The average ratio Vol_V_/Vol_E_ differs significantly*^,^ ** between *S*. *hippolytes* and *Peltogaster* including *Peltogaster* sp. 2 (p = 0.0248) and between *S*. *hippolytes* and *Peltogaster*, without *Peltogaster* sp. 2 (p = 0.0039) (Table 2).

However, Vol_V_ does not differ significantly between *Peltogaster* spp., with and without *Peltogaster* sp. 2 (p = 0.47). The ratio Vol_I_/Vol_E_ is bigger in *Peltogaster* sp. 2 than in *S*. *hippolytes* specimen 1b (Table 1). In other words, the externa of *S*. *hippolytes* is larger in relation to the interna than the externa of *Peltogaster* sp. 2.

### Number of eggs and volume measurements between the eggs and the host

The average number of N_EE_ and N_CE_ differs significantly *^,^ **^,^ * between *S*. *hippolytes* and *Peltogaster* spp. (for N_CE_ p < 0.039, for N_EE_ p < 0.039), between *S*. *hippolytes* exclusive *specimen* 1c and *Peltogaster* (for N_CE_ p = 0.0082, for N_EE_ p = 0.0088) and between *S*. *hippolytes* exclusive *specimen* 1c and *Peltogaster* exclusive *P*. *boschmai* (for N_CE_ p = 0.028, for N_EE_ p = 0.029) (Table 3). The average number of eggs does not differ significantly between N_EE_ and N_CE_ for *S*. *hippolytes* (p = 0.99), for *Peltogaster* (p = 0.99) and for all measured rhizocephalans (p = 0.99).

**Table 3:** Number and volume of estimated and counted parasite’s eggs. The following parameters are results for three individual specimen of *Peltogaster* and five individual specimen of *Sylon*, therefore, no error estimate is possible for each individual.

Specimen	Host	N_EE_	N_EE_ (mean ± standard variation)		N_CE_	N_CE_ (mean ± standard variation)		(Vol_AE_/Vol_H_)×10^−6^	(Vol_AE_/Vol_H_)×10^−6^ (mean ± standard deviation)
*P*. *boschmai*	*D*. *schmitti*	357.44	2,078 ± 2,272		371	2,060 ± 2,224		79.59	29.68 ± 43.23
*P*. *curvata*	*P*. *prideaux*	4,654.05		2,939 ± 2,425	4,580		2,905 ± 2,369	4.33	
*Peltogaster* sp. 1	*P*. *hirsutiusculus*	1,223.74			1,230			5.12	
*S*. *hippolytes* 1a	*P*. *brevirostris*	11,522.74	12,951 ± 8,510	15,929 ± 6,413	11,572	12,954 ± 8,454	15,835 ± 6,321	3.25	2.756 ± 2.46
*S*. *hippolytes* 2a		9,240.69			9,361			0.59	
*S*. *hippolytes* 1b		22,240.67			22,237			1.14	
*S*. *hippolytes* 2b		20,336.33			20,169			2.03	
*S*. *hippolytes* 1c		1,416.25			1,430			6.77	

#### Correlation between different parts of parasites and respective hosts

**Fig 4:**
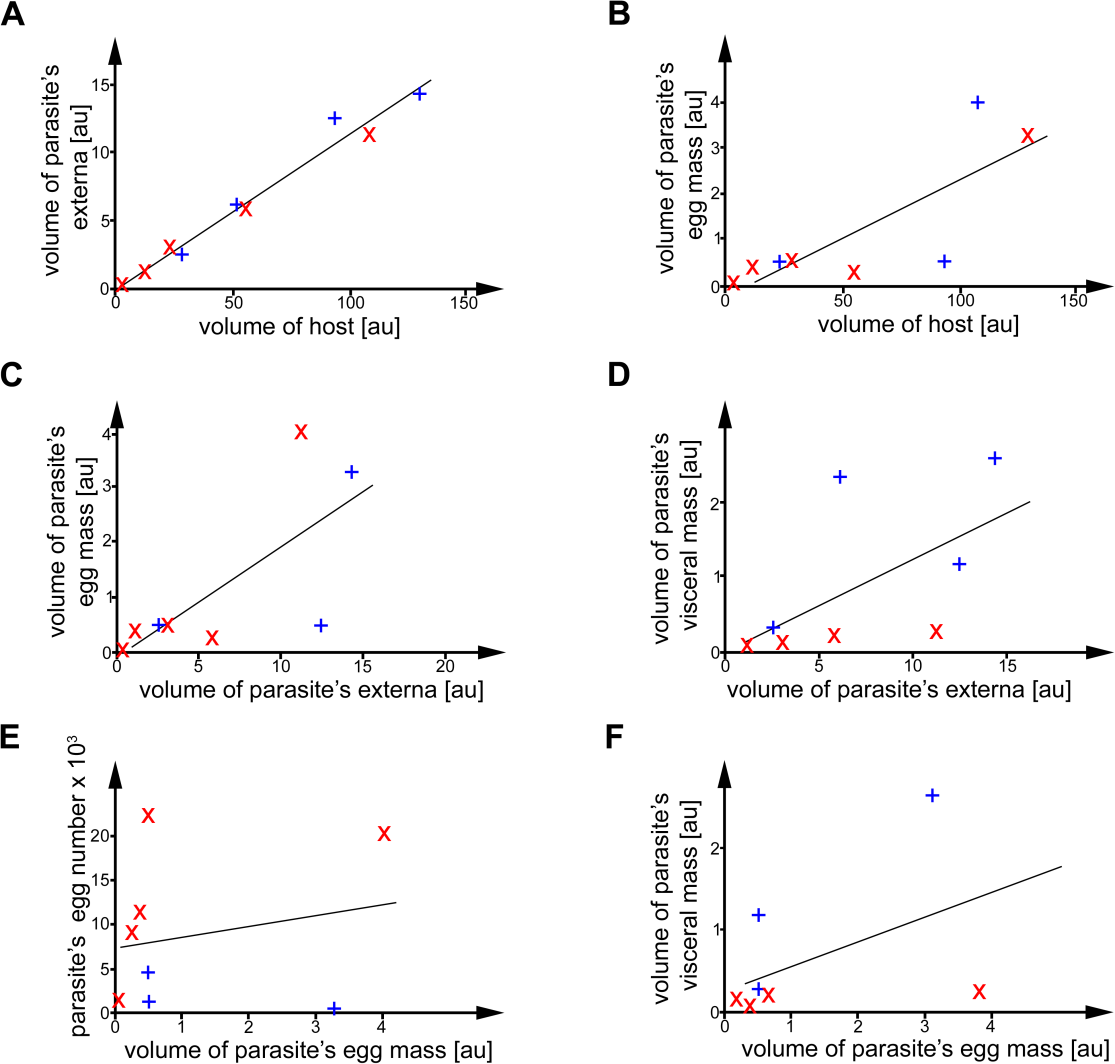
Scatter plots with trend lines of correlations between volume measurements of different parts of the parasite and the host. x = *Sylon* spp., + = *Peltogaster* spp., au = artificial units, the trend lines were computed by least-square linear regression. (A) Volume of parasite’s externa and volume of host. (B) Volume of parasite’s egg mass and volume of host. (C) Volume of parasite’s egg mass and volume of parasite’s externa. (D) Volume of parasite’s visceral mass and volume of parasite’s externa. (E) Parasite’s estimated egg number and volume of parasite’s egg mass. (F) Volume of parasite’s visceral mass and volume of parasite’s egg mass.

The volume of the parasite’s externa increases significantly with the host’s volume (r = 0.98, N = 9, p < 0.001; Fig 4A). The volume of the parasite’s egg mass increases significantly with the host’s volume (r = 0.80, N = 8, p < 0.01; Fig 4B). The volume of the parasite’s egg mass increases significantly with the volume of the parasite’s externa (r = 0.7, N = 8, p < 0.05; Fig 4C). The volume of the parasite’s visceral mass increases significantly with the volume of the parasite’s externa (r = 0.59, N = 8, p < 0.05; Fig 4D). There is no significant correlation between the parasite’s egg number and the volume of the parasite’s egg mass (r = 0.2, N = 8, p < 0.5; Fig 4E). There is no significant correlation between the volume of parasite’s visceral mass and the volume of parasite’s egg mass (r = 0.47, N = 7, p<0.5; Fig 4F).

## Discussion

### Parasite-host-volume-ratio

The body size of parasitic castrators in relation to the body size of their hosts can be used to distinguish between different types of host-parasite interactions [16]. Parasitic castrators are supposed to embody 3–50% of the volume of the host depending on the host and parasite species [15, 25]. Parasitic castrators are defined by absorption of the reproductive effort of the host [14]. The size of an animal matches with its energetic needs [26–28] and the interactions between the parasite and particular host features determine the correlation between parasite and host size [29]. Therefore, since rhizocephalans are parasitic castrators or rather sterilizers, it is reasonable to assume that they occupy a volume of the host that corresponds to the volume of the reproductive organs occupied in a sexually mature but non-infected host [13]. For female decapods in general the reproductive effort has been estimated with 12–25% of their body mass [30], for caridean shrimps 6.9–30.0% [31, 32] and for hermit around crabs 16% [33]. Our results for the volume of the entire parasite, 17.78% for *Peltogaster* sp. infesting *P*. *pubescens* (Fig 3F) and 18.07% for *S*. *hippolytes* infesting *P*. *brevirostris* (Figs 2 and 3I) confirm the estimations made by Lafferty and Kuris [13] for rhizocephalans.

Although an earlier study by Poulin and Hamilton [34] showed no correlation between externa size and host size for rhizocephalans infesting decapods, the majority of studies [27, 28, 35–37] assumed a positive correlation. Our results confirm a significant strong positive correlation for the volume of the externa to that of the host (Fig 4A). A positive correlation between body size and fecundity has been reported for different crustacean groups, e.g. Ascothoracida, Branchiura, Caridea, [34, 38–42]. The reproductive organs grow in a positive allometric proportion to the body size in crustaceans [26], just as rhizocephalans do in a positive correlation to their hosts. The rhizocephalan *Heterosaccus dollfusi* grows in positive allometric proportion to its host the brachyuran *Charybdis langicollis* [43], in the same way *S*. *hippolytes* grows in positive allometric proportion to different species of Pandalidae [35]. We confirm this growth pattern for *S*. *hippolytes* and *Peltogaster* spp. studied herein by our analysis (Figs 3 and 4A). In other words: the bigger the host, the bigger the rhizocephalan. This phenomenon is also known as Harrison`s rule [44, 45]. Harrison’s rule is common among a diverse assemblage of parasites, including parasitic worms, fleas, lice and ticks, as well as in herbivorous aphids, trips, beetles, flies, moths and flower mites [34, 46–50]. A positive allometry has also been reported for other parasitic castrators, e.g. twisted wing parasites and horsehair worms [12, 51–53]. The positive correlation between the volume of the parasite and the volume of the host, found in the present study, confirms that Harrison’s rule can be applied for Rhizocephala and is driven by the reproductive effort of the host.

### Parasites egg and visceral mass

An externa of *S*. *hippolytes* produces only one brood during its lifetime and it has been estimated that this single brood contains between 18,900 and one million eggs, when the rhizocephalan was parasitizing shrimps, e.g. *Spirontocaris liljeborgi* or *Pandalus platyceros*, respectively [35, 36]. After releasing the identical male and female cyprids [36, 54, 55], the externa falls off, and leaves a scar on the abdomen of the host shrimp [35, 56]. The entire lifespan of *S*. *hippolytes* has been estimated to be maximum one year [28]. However, the number of eggs measured for *S*. *hippolytes* in this study (1,430 in specimen 1c (Fig 3E), and from 9,361 in specimen 2a (Fig 3D) to 22,237 in specimen 1b (Figs 2 and 3I)) differs from the previous statements. Considering the size difference between the hosts, with the herein studied *P*. *brevirostris* being smaller than *S*. *liljeborgi*, studied by Lützen [35], and the positive correlation between the number of eggs and the size of the host, it seems that bigger hosts are parasitized by rhizocephalans that carry more eggs. In summary the number of eggs of *S*. *hippolytes* studied herein can range from 1,400 to 22,000 eggs when parasitizing smaller hosts (e.g. *P*. *brevirostris*) and from 19,000 to one million eggs when parasitizing bigger hosts (e.g. *S*. *liljeborgi*).

In contrast, representatives of kentrogonid Peltogastridae hatch as nauplii and have been reported to live as long as five years [28, 56]. Numbers of ovipositions vary between three and five for *Peltogaster paguri* [57] and up to 11 for *Peltogaster curvata* [28]. An egg number between a few hundred and 28,000 has been estimated for *Peltogaster paguri* in one brood [27, 57–61]. Due to the sexual dimorphism reported for rhizocephalans with a kentrogonid lifestyle [62, 63], two different egg sizes (small female eggs, bigger male eggs) occur within the peltogastrids and they show three different types of broods: pure female eggs, mixed female and male eggs and pure male eggs. Therefore, brood composition will have an impact on the number of eggs per volume of egg mass, and subsequently the total offspring of a parasite.

The high variation in number of offspring between the different species of *Peltogaster* in this study, ranging from only 371 eggs in the small externa of *P*. *boschmai* (Fig 3A) to 4,580 eggs in the larger *P*. *curvata* (Fig 3H), illustrates the large impact of host size on the reproductive output of the parasite. This trend is further highlighted in the king crab rhizocephalan *Briarosaccus*, which is closely allied to *Peltogaster* [64]. This parasite, which reaches enormous sizes for rhizocephalans [65, 66], has been reported with up to 500,000 larvae being released in one single spawning event [67].

Akentrogonida releases less cyprids than Kentrogonida releases nauplii [55]. This would be true for our results if multiplying the number of eggs with the assumed ovipostions in *Peltogaster*. For the supposed range of the number of eggs (in *S*. *hippolytes* 15,000–1,000,000 per brood, in Peltogastridae 200–28,000 per brood), we cannot support this statement.

As reported for other crustaceans [68–73] and estimated also for rhizocephalans [61], body size is positive correlated with the number of eggs. Our results provide a slightly positive correlation between the number of eggs and the volume of the egg mass and visceral mass (Fig 4E and 4F). It has been postulated that egg size in Rhizocephala is more or less constrained and the fecundity simply increases with body size [34]. For specimens studied herein, our results give evidence for a strong positive correlation between the externa and the visceral mass (Fig 4D), but just a slightly positive correlation between the egg mass and the egg size (Fig 4E). Additionally, there is no significant relation between rhizocephalan egg volume and volume of the host (Table 3). Therefore, the correlation between the egg number and egg mass might be an artifact (Fig 4E and 4F).

Surprisingly, the volume of the visceral mass, the egg generating tissue, in *S*. *hippolytes* is more constrained than in Peltogastridae (Fig 4D). In *S*. *hippolytes* studied herein the volume of the visceral mass is around 2.5% of the volume of the externa, whereas in *Peltogaster* sp. studied herein it is around 19.5%. *Peltogaster*, which produces multiple broods [57] (Table 2), apparently has more generative tissue than *S*. *hippolytes*, which produces only a single brood. Due to the fact, that representatives of Peltogastridae infest the host for a longer period, they have more time to grow [15]. The limited space inside the host causes a fixed size relation between the parasite and the host. Thus, the parasites are just able to utilize the reproductive energy of the host [13, 15]. In contrast to *S*. *hippolytes*, representatives of *Peltogaster* need to reuse the visceral mass to produce several broods throughout its lifetime and need more energy to produce the larger female eggs and the much larger male eggs.

However, the positive correlation between the egg mass and the volume of the externa (Fig 4C), also leads to a positive correlation between the number of eggs and parasite`s body size. Although earlier studies [34] could not find a correlation between body size and egg size, they assumed a correlation between fecundity and body size. Cavaleiro & Santos [74] have supposed, that this correlation is related to the positive correlation between female body size and ovary size. The positive correlation in our study between the volume of the externa and the volume of the visceral mass, largely containing the ovaries, supports this hypothesis (Fig 4D). Assuming that the fecundity of a parasite is proportional to its body size and the parasite size is proportional to the host size, the host size represents an indicator for the fecundity of its parasite.

### Two different lifestyles and phylogenetic interpretation

The different life traits of the two investigated groups can be interpreted in terms of an r/K-continuum [75]. r-Strategists have a rapid development, and often small body size and a high rate of reproduction together with a large reproductive effort and environmental uncertainty [76, 77]. In contrast, K-strategists show a delayed, sexual maturity, often large body size, small number of offspring and a smaller reproductive effort, steady environmental conditions, sexual dimorphism with bigger males [76–78].

Interestingly, intra-species competition has been proposed to be higher in K-strategists [78, 79]. The higher intraspecific competition in K-strategists can cause the migration behavior of infested crabs into deeper waters, where the competition for nutrients is less severe [80–84], because the host hermit crabs act as the extended phenotype of peltogastrid rhizocephalans [12, 13, 25].

The lack of naupliar stages in akentrogonids increases the survival success of cyprid stages by reducing the risk of predation in the planktonic stages, but decreases the dispersal ability [55, 61]. According to Høeg [55] the shortened free larval life span can be seen as a specialization for remaining in the home range of a host population of non-stationary hosts and, therefore, a higher survival rate of the akentrogonids. Rhizocephalans with a kentrogonid lifestyle compensate for the larval loss by increasing the lifetime reproductive success of individual females, producing several broods with morphologically different nauplii that have a better chance of reaching areas with new hosts [55, 74, 85]. Due to multiple broods and continuation of growth in kentrogonid externae, externa molting between broods is an integrated part of an adult parasite. In most, but not all representatives of Akentrogonida, on the other hand, the externa produces only a single brood of larvae and molting is not required [28].

Characters that indicate that *S*. *hippolytes* leans more towards an r-strategy than the peltogastrids studied herein, are 1) the lack of naupliar stages and faster larval development, 2) the lack of sexual dimorphism in the body size of female and male cyprids, 3) the larger egg numbers per brood (about 13,000 in *Sylon* spp. vs. about 3,000 in *Peltogaster* spp.) 4) the smaller volume of the visceral mass (when oviposition has taken place) (about 2.5% in *Sylon* spp. vs. about 19.5% in *Peltogaster* spp.) and, 5) the smaller average egg size (0.003‰ of host size in *Sylon* spp. vs. 0.005‰ of host size in *Peltogaster* spp.).

Concomitantly, rhizocephalans generally show a high degree of host-specificity [12, 13, 86]. Although host-specificity may not be limited to a single species, they show host-specificity at a higher systematic level. *S*. *hippolytes* has been reported to parasitize 26 species of caridean shrimp [36, 57, 87], however the species might be a complex of cryptic species with higher host specificity. Representatives of *Peltogaster* have been reported to parasitize hermit crabs (Paguridae, Diogenidae) [88, 89]. In comparison to other parasitic castrators, rhizocephalans show a broader host range [90]. Based on physiological studies, it is likely that rhizocephalans parasitize hosts within their species-specific host range that inhabit a preferred habitat [91–93].

Based on recent studies [5, 86, 94] the evolutionary key events for the rhizocephalans seem to have been: 1) parasitism of Anomala *sensu* Scholtz & Richter [95] by an infective kentrogon stage, succeeding the cypris larval stage, 2) parasitism of brachyurans, 3) parasitism with great modifications (loss of the kentrogon stage and the reduction of larval life span due to the reduction of the nauplius larval stage), 4) this modified akentrogonid morphology apparently opened for a broader range of hosts across decapods and other crustaceans, via host switches between distant related groups. The transformation from the kentrogon penetration method to the akentrogonid penetration method occurred just in a single evolutionary event [1] and evolved likely synchronous with a more r-strategic life history (Fig 1). In evolutionary terms, rhizocephalans have been successful by adopting different parasitic modes of life, and explored most evolutionary possibilities by reducing their morphological characters to a minimum.

### Methodological notes

This manuscript should show an easy way to reconstruct the interna of *S*. *hippolytes* by injecting iodine directly into the externa prior scanning with a micro-CT. The data from the CT should then be analyzed just by using the different grey values of the tissue between the host and the parasite. To emphasize this method, we visualized our results on the example of *S*. *hippolytes* specimen 1b (Figs 1 and 2I). Unfortunately, other staining methods like phosphotungzid acid did not achieve enough contrast between the rhizocephalan interna and the internal structures of the host. Some structures, e.g. visceral mass, are not visible or even lose their shape (*S*. *hippolytes* specimen 1c, Fig 3E). Furthermore, we explain, that this method does not allow any replications in measuring the volumes of specific parts, e.g. host, parasite’s externa, parasite’s interna, parasite’s egg mass, parasite’s visceral mass, because the software (Osirix and Blender) will use always the same algorithm. To achieve statistically more powerful analyses, we have to study more specimens.

However, staining with iodine (directly injected or the deposition of the specimens in iodine) achieved a high contrast between the cuticle of the rhizocephalan externa, the rhizocephalan eggs and the rhizocephalan visceral mass, at least in eight of nine specimens. In contrast to the conventional method of examination of the externa, which requires the destruction of the specimens (histological sectioning, dissections), this new method serves as a non-disruptive and fast alternative [7]. Thus, the method described herein can be used for further fast estimation of the life history and investigation of the morphology in other rhizocephalan species.

## Conclusion

We could show

- a positive correlation between the body size of *S*. *hippolytes* and the body size of their hosts
- a positive correlation between the body size of *Peltogaster* spp. and the body size of their hosts
- a positive correlation between the volume of the externa of *S*. *hippolytes* and *Peltogaster* and the volume of the body of their hosts
- a positive correlation between the volume of the visceral mass and the volume of the body of the rhizocephalans studied herein
- a positive correlation between the volume of the egg mass and the volume of the externa of rhizocephalans studied herein
- a positive correlation between the egg number and the egg mass of rhizocephalans studied herein.

Furthermore, it was possible to map the life history traits of the specimens studied herein on their phylogenetic tree (Fig 1). This study provides evidence that the akentrogonid *S*. *hippolytes* shows more r-strategic characters than the studied representatives of Peltogastridae with a kentrogonid lifestyle. Studying the extremely host-exploiting (the parasite exerts a very high energetic cost on the host) *Sacculina carcini* [57] may yield surprises about the life history traits and the general evolution of parasitism within Rhizocephala. This study has added to our global understanding of the evolution of parasitic castrators within Rhizocephala and the different parasitic strategies within parasitic castrators.
